# Rapid Quantification and Validation of Lipid Concentrations within Liposomes

**DOI:** 10.3390/pharmaceutics8030029

**Published:** 2016-09-13

**Authors:** Carla B. Roces, Elisabeth Kastner, Peter Stone, Deborah Lowry, Yvonne Perrie

**Affiliations:** 1 Strathclyde Institute of Pharmacy and Biomedical Sciences, University of Strathclyde, Glasgow G4 0RE, UK; carla.roces-rodriguez@strath.ac.uk; 2 Aston Pharmacy School, School of Life and Health Sciences, Aston University, Birmingham B4 7ET, UK; kastnere@aston.ac.uk (E.K.); stonep@aston.ac.uk (P.S.); 3 School of Pharmacy and Pharmaceutical Sciences, Ulster University, Coleraine BT52 1SA, UK; d.lowry@ulster.ac.uk

**Keywords:** lipids, liposomes, cholesterol, quantification, HPLC, ELSD

## Abstract

Quantification of the lipid content in liposomal adjuvants for subunit vaccine formulation is of extreme importance, since this concentration impacts both efficacy and stability. In this paper, we outline a high performance liquid chromatography-evaporative light scattering detector (HPLC-ELSD) method that allows for the rapid and simultaneous quantification of lipid concentrations within liposomal systems prepared by three liposomal manufacturing techniques (lipid film hydration, high shear mixing, and microfluidics). The ELSD system was used to quantify four lipids: 1,2-dimyristoyl-*sn*-glycero-3-phosphocholine (DMPC), cholesterol, dimethyldioctadecylammonium (DDA) bromide, and d-(+)-trehalose 6,6′-dibehenate (TDB). The developed method offers rapidity, high sensitivity, direct linearity, and a good consistency on the responses (*R*^2^ > 0.993 for the four lipids tested). The corresponding limit of detection (LOD) and limit of quantification (LOQ) were 0.11 and 0.36 mg/mL (DMPC), 0.02 and 0.80 mg/mL (cholesterol), 0.06 and 0.20 mg/mL (DDA), and 0.05 and 0.16 mg/mL (TDB), respectively. HPLC-ELSD was shown to be a rapid and effective method for the quantification of lipids within liposome formulations without the need for lipid extraction processes.

## 1. Introduction

Liposomes continue to be a major focus in drug delivery research due to their ability to enhance the delivery and targeting of a wide range of drugs and vaccines. In the majority of products approved for clinical use, a combination of a phosphatidylcholine and cholesterol is used, with some examples outlined in Table 1. However, as new applications are developed, and new lipids become available, we are able to exploit a vast range of lipid combinations so as to further enhance the efficacy of these formulations. For example, recent collaborative work from our laboratories has focused on the use of cationic liposomal adjuvants to enhance the delivery and efficacy of sub-unit antigens. The adjuvants are based on a two-component vesicle formulation, which is the combination of the cationic lipid dimethyldioctadecylammonium (DDA) and d-(+)-trehalose 6,6′-dibehenate (TDB). DDA is a synthetic amphiphile discovered to have adjuvant properties by Gall in the mid-1960s [1]. It contains a quaternary ammonium group with two 18-carbon-long alkyl chains (forming the hydrophobic moiety) and two methyl groups, which, together with the ammonium group, form the polar head group. The positively-charged head group carries a monovalent counterion, typically bromide or chloride. TDB is a synthetic analog of trehalose-6,6-dimycolate, an immunostimulatory component of *Mycobacterium tuberculosis*. It has two 22 carbon saturated fatty-acid chains (behenyl), each replacing the branched mycobacterial mycolic acids of >70 carbons. These two behenyl chains are linked by ester bonds to carbon number six of each of the two glucopyranose rings making up the trehalose head group. TDB has been shown to retain much of the bioactivity of the native form, while showing less toxicity as a result of the shorter fatty acid chains [2,3]. Formulation of vesicles from DDA alone produces vesicles able to induce cell-mediated immunity and delayed-type hypersensitivity. Along with its cationic nature and surfactant properties, it has been shown to be an effective adjuvant in numerous applications, including mucosal immunization [4], gene delivery [5,6], and subunit vaccine delivery [7,8,9]. However the combination of DDA and TDB within the liposome formulation was shown to further enhance the adjuvant properties of the liposomes [10]. This lipid combination is known as the cationic adjuvant formulation (CAF) 01.

**Figure 1:**
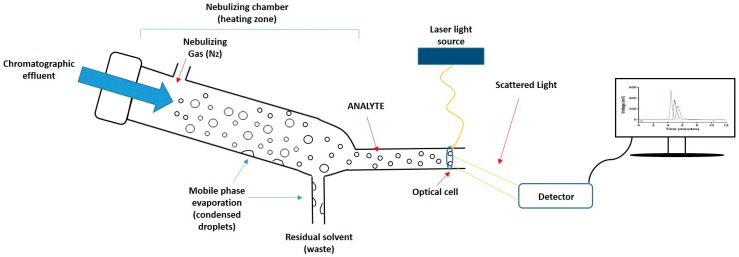
Schematic representation of the mechanism of an evaporative light scattering detector (ELSD). First, the chromatographic eluate passes through the nebulizer and mixes with the nebulizing gas (N_2_), causing dispersion of droplets; subsequently, droplets enter into the nebulizing chamber where the mobile phase evaporates and condenses, being removed as waste; Finally, the analyte crosses an optical cell, a laser beam penetrates the particles, and the scattered light is detected and converted into a signal.

Given the importance of lipid composition in liposome formulations in terms of their drug incorporation and release properties, pharmacokinetic properties, and their stability, the quantification of lipid components is a key product attribute. However, the development of rapid and simple quantitative tools for the analysis of lipids has been slow. This is primarily due to the absence of easily detectable functional groups (e.g., chromophores) on most lipids, which makes them difficult to measure quantitatively using the more common types of detectors. Therefore, while high-performance liquid chromatography (HPLC) is the technique of choice for the efficient separation of lipid components based on different chain lengths, head group composition, and polarities [11,12,13,14,15,16], appropriate detectors for lipids are required for the quantification of these lipids upon separation. To achieve this, mass detectors such as evaporative light scattering detectors (ELSD) can be used [17]. ELSD is used for the quantification of non-volatile components dissolved in volatile solvents. In this method, a nebuliser evaporates the solvent (mobile phase) inside the heating chamber. Solvent droplets condense within the chamber and are removed from the system. Meanwhile, the non-volatile compound (analyte) becomes a mist of small particles which continue moving, by means of a stream of compressed air, towards the detector. When sample particles pass through the optical cell, a laser beam illuminates the sample, and when the laser hits the particles, the intensity of the scattered light is measured by a detector (Figure 1). The detector can quantify the amount of sample according to the scattered light detected. This method is constrained to a sufficiently volatile solvent with a different volatility than the analyte, and for the separation of lipid classes, gradient elution is often required.

In addition to considering quantification, lipid degradation is also an important factor that needs to be considered. The main route of phospholipid degradation is hydrolysis [18]. This can occur rapidly, and can be promoted by increases in temperature and/or harsh processing conditions, such as sonication. However, the use of bath sonication may circumvent this issue [19]. The products of the hydrolysis of phospholipids are lysolipids, and they have a detergent effect which can interfere with the liposome bilayer. This is due to the lysolipids stabilizing pores in the membrane, thereby enhancing the permeability of the bilayer and promoting liposome bilayer fusion [20,21]. This process can occur even at low lysolipid concentrations [22]. Lipids can also be susceptible to degradation via oxidation of polyunsaturated hydrocarbon chains and ester hydrolysis, producing oxidized lysophosphatide and free fatty acid derivatives. These products of oxidation also have an effect on membrane permeability [23]. However, it has been shown that the presence of cholesterol within a liposome formulation can reduce oxidation by reducing lipid-bilayer hydration [24]. To quantify degradation, previous work has centered around using NMR and mass spectrometry to analyze the products of lipid degradation; however, these methods can be both time consuming and expensive to run [25].

Given that the choice of lipid composition directly impacts liposome efficacy and stability, the aim of this current study was to develop and validate a rapid quantification method for lipids that does not require an initial lipid extraction process. This method was used to quantify lipids within the DDA:TDB liposome adjuvant system and also to demonstrate the general procedures such that they can be applied to a wide range of lipids used by liposomologists. As such, we demonstrate the quantification of four lipids using HPLC-ELSD: DDA, TDB, DMPC, and cholesterol, with a detailed focus on cholesterol given that it is the most common component of liposome systems. We also consider the ability of this HPLC-ELSD method to detect phospholipid lipid degradation.

## 2. Materials and Methods

### 2.1. Materials

The cationic surfactant dimethyldioctadecylammonium (DDA) bromide, neutral lipid 1,2-dimyristoyl-*sn*-glycero-3-phosphocholine (DMPC) and the immunopotentiator α,α′-trehalose 6,6′-dibehenate (TDB) were obtained from Avanti Polar Lipids Inc., Alabaster, AL, US. Cholesterol was obtained from Sigma-Aldrich Company Ltd., Poole, UK. 2-amino-2-(hydroximethyl)-1,3-propanediol (Trizma base^®^) was obtained from ICN Biomedicals Inc., Aurora, OH, US, and ultrapure water was obtained from a Milli-Q purification system (Millipore, Billerica, MA, US). All other reagents, such as chloroform, methanol, isopropyl alcohol, phosphate-buffered saline (PBS), and trifluoroacetic acid (TFA) were of analytical grade and purchased from commercial suppliers.

### 2.2. Manufacturing of Liposomes

Liposomal formulations were prepared in triplicate (*n* = 3) by using three different methods. With the traditional lipid film hydration (LH) method [26] weighed amounts of DDA (10 mg/mL), TDB (2 mg/mL), cholesterol (2 mg/mL), and DMPC (10 mg/mL) were dissolved in a mixture of chloroform:methanol (*v/v* 9:1) for the preparation of the stock solutions. The required amount of lipid solution was transferred to a round-bottom flask to reach the desired lipid concentration and mixed; DDA and TDB concentrations were fixed at 2.5 and 0.5 mg/mL, respectively, and cholesterol was added to the CAF01 formulation at a concentration of 0.8 mg/mL. In the third formulation, DDA was replaced by DMPC and kept at the original concentration. Organic solvent was removed under vacuum with a rotary evaporator for 15 min at 200 revolutions per minute (rpm) at 37 °C in a water bath. Afterwards, the lipid film was dried with a gentle stream of N_2_ in order to remove any trace of organic solvent. Then, the lipid film was hydrated with 1 mL of 10 mM Tris buffer (pH 7.4) at 60 °C (DDA has the highest *T*_m_ (~47 °C) of all four lipids; thus, hydration of the lipid film was at 10 °C above it) with vortexing every 5 min for 20 min.

After preparation by the thin lipid film, the formed liposomes were subjected to probe sonication (MSE Soniprep 150 plus with MSE probe 100, MSE, London, UK) at an amplitude of 10 amps. The liposomes were sonicated for 10 min. The liposome samples were placed in 1.5 mL Eppendorf tubes and then subjected to centrifugation (Grant-bio PCV-3000, Grant, Shepreth, UK) at 1500 rpm for 20 min to remove any large contaminants from solution. Supernatant (100 µL) was collected for size analysis.

The thin film method followed by high shear mixing (HSM) (SilentCrusher M, Heidolph instruments, Schwabach, Germany) [27]: after preparation of the thin lipid film, formation of liposomes was carried out by hydration of the film with 1 mL of Tris buffer (10 mM, pH 7.4) and high shear mixing at 60 °C. The head of the HSM was immersed in the formulation, and samples were homogenized at high speed (240,000 rpm) for 15 min.

Microfluidics (MF) (Nanoassemblr™ Benchtop, Precision Nanosystems Inc., Vancouver, BC, Canada) [28]: in order to produce DDA or DMPC-containing liposomes, lipids were dissolved in the organic solvent (isopropyl alcohol) at the concentrations mentioned above. Organic and aqueous phase (Tris buffer 10 mM, pH 7.4) were heated to 60 °C before and during injection into the system. Control parameters of the microfluidics system were varied in order to obtain the smallest liposome size. After liposome preparation, samples were dialyzed (membrane cut off 3500 Da, Medicell Membranes, Ltd., London, UK) against 200 mL of Tris buffer during 1 h in order to remove the organic solvent.

### 2.3. HPLC Method

Quantification of the lipid recovery was performed by reverse phase high performance liquid chromatography (HPLC) (YL instrument, Anyang, Korea) using a SEDEX 90LT ELSD detector (Sedex Sedere, Alfortville, France) connected to the instrument. The use of evaporative light-scattering detectors can result in limited direct linearity, and when larger concentration ranges are required, a standard log–log transformation can be used. However, within our studies, we use the SEDEX 90LT, which offers a notably increased overall direct linearity range. A Phenomenex Luna column 5 µm C18(2) 4.60 mm inner diameter and 150 mm length with 100 Ă pore size (Phenomenex, Macclesfield, UK) was used as stationary phase, since a C18 column increases the retention of non-polar analytes. Separation of the lipids was carried out using an elution gradient analysis displayed in Table 2. Mobile phase A consisted of 0.1% TFA in water, whereas mobile phase B consisted of 100% methanol. Standard lipid solutions were dissolved in chloroform:methanol (9:1 *v/v*) prior to injection, whereas liposome formulations were injected after manufacturing with no prior modification. The HPLC-ELSD settings were kept constant as follows: 30 µL injection volume in a partial loopfill injection mode, 100 µL loop volume and 15 µL tubing volume. Column temperature was maintained at 35 °C, whereas the ELSD temperature was set at 52 °C in all the runs. Nitrogen was used as a carrier gas at 3.5 psi inlet pressure. Chromatograms were analyzed with Clarity DataApex version 4.0.3.876 software.

### 2.4. Standard Solutions

Standard solutions of cholesterol (0.025 to 1 mg/mL), TDB (0.2 to 1 mg/mL), DDA (0.125 to 2.5 mg/mL), and DMPC (0.25 to 2.5 mg/mL) were prepared in chloroform:methanol (*v/v* 9:1). Solutions were injected into the HPLC system prior to each measurement in order to establish the calibration curves and as a reference check.

### 2.5. Method Validation

The method was validated by assessing the linearity, reproducibility, robustness, accuracy, limit of detection (LOD), and limit of quantification (LOQ). For quantification, established calibration curves were used. The system was flushed with 100% methanol before each use for 30 min until a stable baseline was observed.

### 2.6. HPLC Lipid Degradation Method

Separation of lipid degradation products was carried out using an isocratic flow method (85% methanol and 15% of 0.1% TFA in water) at a flow rate of 1 mL/min allowed the lipids to separate down the column. HPLC-ELSD settings were kept constant as outlined in Section 2.3 using the Phenomenex Luna 5 µm C8(2) 4.60 mm inner diameter and 150 mm length with 100 Ă pore size. The specificity of the method was tested by conducting forced degradation of a liposome formulation using 0.1N HCl at room temperature for four days, as previously described by Zhong and Zhang [29].

## 3. Results and Discussion

### 3.1. Lipid Quantification Using HPLC-ELSD: Method Development

**Figure 2:**
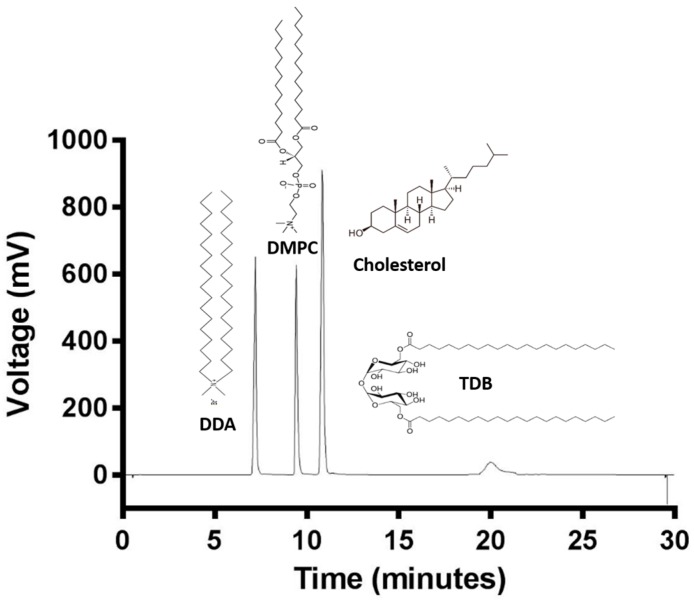
ELSD-detected HPLC chromatogram of DDA (1.5 mg/mL), DMPC (1.5 mg/mL), Cholesterol (2 mg/mL), and TDB (1 mg/mL).

**Figure 3:**
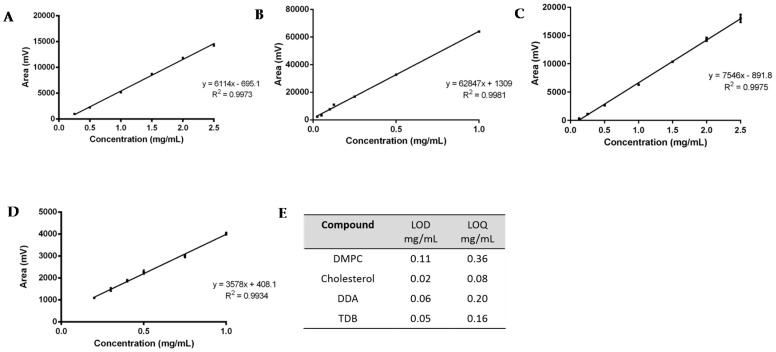
Linearity assessment of (**A**) DMPC (0.25 to 2.5 mg); (**B**) Cholesterol (0.025 to 1 mg); (**C**) DDA (0.125 to 2.5 mg); and (**D**) TDB (0.2 to 1 mg). The LOD and LOQ for each lipid is shown in (**E**). Results represent mean ± SD of triplicate measurements (*n* = 3).

The cationic lipid DDA, and other neutral lipids such as cholesterol, DMPC, and the immunopotentiator TDB are common compounds of liposomal adjuvant formulations. Lipid quantification is of great importance for the stability and efficacy of liposomal formulations. Therefore, an HPLC-ELSD method for the quantification of these lipids was developed in order to achieve the analysis of all four lipids at the same time. Figure 2 shows the chromatogram of a mixture of the four lipids and their chemical structures. DDA is the least hydrophobic lipid and eluted at 7.1 min, followed by DMPC at 9.2 min, cholesterol at 10.9 min, and finally TDB at 19.9 min due to its hydrophobicity. Analysis time was set at 35 min, even though all four lipids eluted within the first 20 min, in order give time to the column to equilibrate. Elution peaks were well separated with a stable baseline. TDB peaks were short and wide, whereas DDA, DMPC, and cholesterol peaks were high and sharp. Chromatographic methods have to be tested in order to guarantee trustworthy and solid data. Therefore, validation of the HPLC-ELSD method was performed according to the International Conference of Harmonization (ICH) guidelines Q_2_ (R_1_) [30] in terms of linearity, sensitivity, accuracy, reproducibility, and robustness, and as an example, a complete validation protocol for cholesterol is outlined in Figure 3.

For the linearity assessment (Figure 3A–D), standard solutions of each lipid were prepared in chloroform:methanol (9:1 *v/v*). A different concentration range was chosen for each lipid, based on the initial concentration used for the preparation of liposomes: for DDA, the concentration ranged from 0.125–2.5 mg/mL; for DMPC, from 0.25–2.5 mg/mL; for TBD, from 0.2–1 mg/mL; and for cholesterol, 0.025–1 mg/mL. The mean peak area ± standard deviation (SD) was calculated and plotted against the known concentration of the standard. ELSDs are known to be non-linear; however, SEDEX 90LT provides direct linearity and a good consistency on the responses. From Figure 3A–D it can be seen that all calibration curves display a good linear fit, with correlation coefficients (*R*^2^) greater than 0.993.

Study of the sensitivity of the method was assessed by means of the calculation of the limit of detection and the limit of quantification (Figure 3E). Values were determined from the standard deviation of the response (σ) and the slope (*S*) obtained from the calibration curves carried out during the linearity assessment. According to the ICH guidelines, a signal-to-noise ratio of three was assumed for the quantification of the LOD, whereas for the LOQ, a signal-to-noise ratio of ten was set. Therefore, following Equations (1)–(3), the sensitivity of the method for the detection of DDA, TDB, DMPC, and cholesterol was calculated.

(1) $$\sigma \;=\;\;\sqrt{\frac{\Sigma \;{\left(Y\;-\;Y_{i}\right)}^{2}}{n\;-\;2}}$$

(2) $$LOD\;=\;\frac{3\sigma }{S}$$

(3) $$LOQ\;=\;\frac{10\sigma }{s}$$

The corresponding LOD and LOQ were 0.11–0.36 mg/mL, 0.02–0.80 mg/mL, 0.06–0.20 mg/mL, and 0.05–0.16 mg/mL for DMPC, cholesterol, DDA, and TDB, respectively (Figure 3E). TDB was the most difficult lipid to quantify, since the intensity of the peaks and the concentration used for the formulations was lower compared to the other compounds, which reflects the concentrations used within liposome formulations.

**Figure 4:**
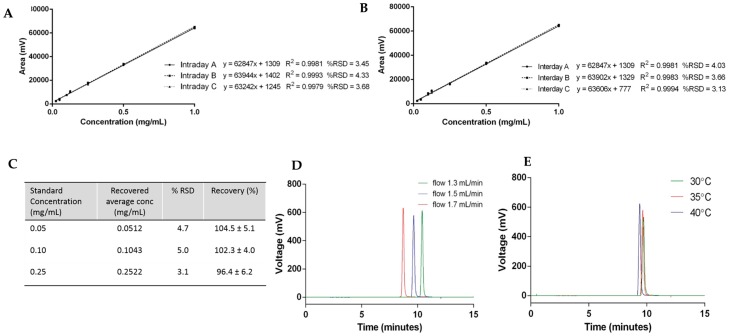
Cholesterol quantification case study: (**A**) intraday variability; (**B**) interday variability; (**C**) accuracy; and robustness with (**D**) flow rate and (**E**) temperature.

The repeatability of the method is the determination of how close values are to each other under identical experimental conditions, and was assessed by determination of the intraday and interday variability using cholesterol (Figure 4). Cholesterol standards from 0.025 to 1 mg/mL carried out within the same day (intraday) and over the course of three days (interday) are plotted in Figure 4A,B, respectively. For the linearity assessment, the average peak area ± SD measurements of each concentration were plotted against the known cholesterol concentration. The results show that the values have no detectable difference for all the calibration curves carried out at different times on the same day and also on different days, meaning the method has good precision.

To test the accuracy of the HPLC-ELSD method, spiked samples of cholesterol standards were prepared and quantified. Figure 4C shows the results of three replicates of cholesterol concentrations at 0.05, 0.1, and 0.25 mg/mL injected into the HPLC-ELSD system in triplicate (*n* = 9). The relative standard deviation (% RSD) and the recovery of cholesterol were calculated for each concentration, and were within the acceptance limit of ≤5% and 90%–110%, respectively (Figure 4C).

Robustness—which represents the ability of the method to remain unchanged by small deviations—was demonstrated by variation in the flow rate and column temperature. To consider this the flow rate, which was initially set at 1.5 mL/min, was changed by ±0.2 mL/min, and Figure 4D displays the elution peaks of cholesterol by variation of the flow rate. Increased flow rate—which represents an increased volume and speed of mobile phase through the column—resulted in a faster elution of cholesterol. In contrast, a decreased flow rate resulted in the later elution of cholesterol from the column. When the column temperature was modified ±5 °C, small variations in the cholesterol elution time and column height were seen (Figure 4E). An increase in temperature accelerates the elution of cholesterol, which can be explained by an increase in the diffusion of the molecules due to the higher temperature. Due to the changes observed during the robustness assessment, flow rate and column temperature were fixed at 1.5 mL/min and 35 °C, respectively.

### 3.2. Quantification of Lipids within Liposomal Adjuvant Formulations

**Figure 5:**
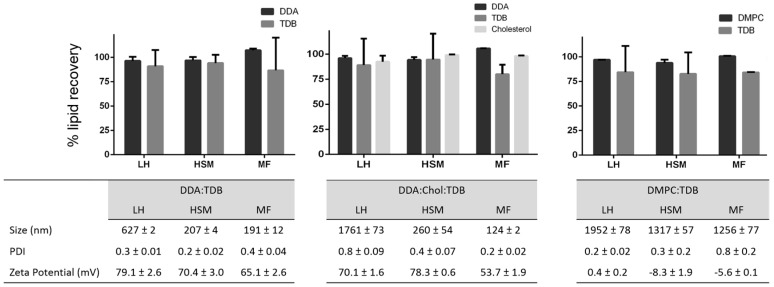
Lipid recovery, size, polydispersity index (PDI) and zeta potential (ZP) from liposomes prepared by lipid hydration (LH), high-shear mixing (HSM), and microfluidics (MF). Results represent the mean ± SD of three replicate batches (*n* = 3).

After method validation, three liposomal formulations were prepared: DDA and TDB concentrations were fixed at 3.96 and 0.5 mM, respectively, and cholesterol was added to this formulation at a concentration of 31 mol % in order to increase the fluidity of the membrane bilayer and favor the formation of liposomes at room temperature. In the third formulation, DDA was replaced by DMPC. These formulations were prepared using three different manufacturing methods. Lipid film hydration is the classical method used to prepare liposomes. It has been used since 1960s, and since then, variation of the method and other methods for liposome preparation have emerged. Among them are the combination of LH with high shear mixing [27] and microfluidics [31,32,33]. Microfluidics manipulates the mixing of fluids in micro-sized channels, allowing for size-controlled liposome preparation [31]. It has several advantages compared to other methods, such as high reproducibility and scalability, low cost, and easy control. However, it is important to consider lipid concentrations post-production of liposomes, given that this can impact the physicochemical characterization of the resulting liposomes and will determine the performance of these delivery systems. The results (Figure 5) obtained from each technique showed that larger liposomes were prepared by the traditional LH method, whereas HSM and MF produced smaller liposomes and more homogeneous particle size distributions, as would be expected with these methods (Figure 5). Similarly, as the same lipid composition was used in each method, the zeta potentials for all three formulations were similar, irrespective of the manufacturing method (Figure 5). Given that the aim of this research was to develop a rapid procedure for the quantification of lipids within liposomes, without the need for extraction processes, formulations were injected into the HPLC-ELSD system, without prior modification, for quantification of the lipid recovery (Figure 5). Lipid quantification revealed a lipid recovery of 96%, 88%, 93%, and 97% for DDA, TDB, cholesterol, and DMPC, respectively, after manufacturing using LH. Liposome formulations manufactured using HSM showed a recovery of 95%, 90%, 99%, and 94% for DDA, TDB, cholesterol, and DMPC, respectively. Finally, microfluidics exhibited a recovery for DDA, TDB, cholesterol, and DMPC of 100%, 83%, 98%, and 100% respectively. For the immunopotentiator TDB, the results were more variable than for the other lipids, but was >80% across the formulations, presumably due to the low concentrations of TDB used in the formulations. In general, while the method of liposome formulation impacted liposome characteristics as would be expected, there are no significant differences in the recovery of the lipids between each of the three methods employed.

### 3.3. The Use of HPLC-ELSD to Assess Lipid Degradation in Liposome Formulations

**Figure 6:**
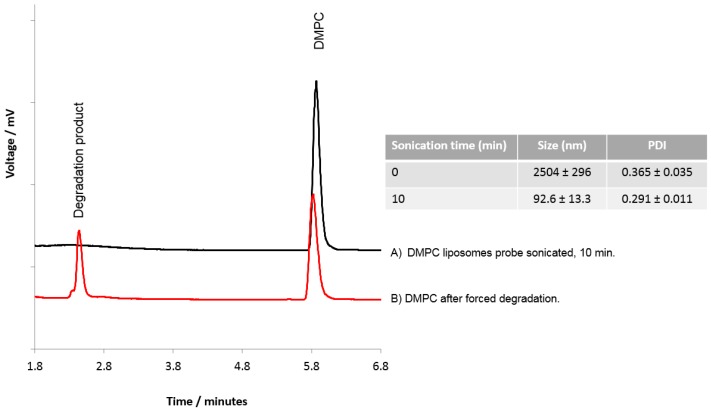
Analysis of lipids within DMPC liposomes. DMPC liposomes were prepared and subjected to (**A**) probe sonication for 10 min, or (**B**) forced degradation using HCl.

To consider if HPLC-ELSD could be used to assess lipid degradation, DMPC liposomes were subject to probe sonication and forced degradation using HCl. Probe sonication of the liposomes for 10 min was shown to reduce the DMPC liposomes to ~90–100 nm with a PDI of 0.291 ± 0.011 (Figure 6), and HPLC-ELSD analysis did not detect any degradation products resulting from sonication (Figure 6A). However, degradation products from DMPC were detected with HPLC after forced degradation (Figure 6B), as represented by a peak at 2.4 min equating to degradation products, as previously shown by Zhong and Zhang [29]. Zhong et al. were able to show that dipalmitoyl phosphatidylcholine (DPPC) can degrade into palmitic acid and the lyso forms of DPPC. From our results, it cannot be confirmed which degradation products we were able to detect; however, the change in the DMPC peak and the formation of a new peak does suggest lipid degradation. While the data presented in Figure 6 suggests that the lipids were resistant to degradation via short-term sonication, previous studies have shown that probe sonication can cause an increase in hydrolysis and peroxidation of the lipids within the liposome bilayer, which can cause liposome instability, enhance the membrane permeability, and promote liposome fusion [19,23,34,35]. For more detailed analysis of lipid degradation, mass spectroscopy would be more beneficial. However, HPLC-ELSD can also offer insight into potential lipid degradation.

## 4. Conclusions

Using HPLC-ELSD, we were able to rapidly and simultaneously quantify lipid concentration within liposomal systems prepared from a range of lipids, and we have validated and applied this method to demonstrate high lipid recovery within liposomes prepared by a range of methods. This demonstrates the applicability of HPLC-ELSD for the rapid quantification of lipids within liposomal systems for drug and vaccine delivery.

## Figures and Tables

**Table 1:** Examples of approved liposome formulations.

Product Name	Drug Delivered	Lipid Composition
AmBisome^®^	Amphotericin B	Hydrogenated soy phosphatidylcholine, cholesterol, disteraroylphosphatidyl glycerol, and α tocopherol
Caelyx^®^/Doxil^®^	Doxorubicin	PEGylated distearoylphosphatidyl ethanolamine, hydrogenated soy phosphatidylcholine, and cholesterol
DaunoXome^®^	Daunorubicin	Distearylphosphatidylcholine and cholesterol
Definity^®^	Octafluoropropane	Dipalmitoylphosphatidic acid, dipalmitoylphosphatidylcholine, and PEG-500 dipalmitoyl phosphatidyletholamine
DepoCyte^®^	Cytarabine	Dioleoylphosphatidylcholine, cholesterol, dipalmitoylphosphatidylglycerol, and triolein
DepoDur^®^	Morphine sulfate	Dioleoyl phosphatidylcholine, dipalmitoyl phosphatidylglycerol, cholesterol, and triolein
Epaxal^®^	Inactivated hepatitis A virus	Phosphatidylcholine, cephalin, and purified virus surface antigens
Inflexal V^®^	Influenza haemagglutinin glycoprotein and neuraminidase	Similar to Epaxal; phosphatidylcholine, cephalin, and purified virus surface antigens
Myocet^®^	Doxorubicin	Egg phosphatidylcholine and cholesterol
Visudyne^®^	Vereporfin	Dimyristoylphosphatidylcholine and egg phosphatidylglycerol

**Table 2:** Gradient elution method for quantitative analysis of cholesterol, 1,2-dimyristoyl-*sn*-glycero-3-phosphocholine (DMPC), dimethyldioctadecylammonium (DDA), and d-(+)-trehalose 6,6′-dibehenate (TDB). TFA: trifluoroacetic acid.

Time (min)	% Eluent A (0.1% TFA in ^d^H_2_O)	% Eluent B (MeOH)	Flow Rate (mL/min)
0	15	85	1.5
6	0	100	1.5
25	0	100	1.5
26	15	85	1.5
35	15	85	1.5

## Supporting Information Available

Data presented in this publication can be found at DOI:10.15129/4338bb40-6f84-4dde-b275-6f1656cc5c05.
